# Pulmonary infection caused by Nocardia cyriacigeorgica in a patient with allergic bronchopulmonary aspergillosis: A case report: Erratum

**DOI:** 10.1097/MD.0000000000014356

**Published:** 2019-01-25

In the article, “Pulmonary infection caused by Nocardia cyriacigeorgica in a patient with allergic bronchopulmonary aspergillosis: A case report”,^[1]^ which appeared in Volume 97, Issue 43 of *Medicine*, the corresponding author email should be wujianyong@zju.edu.cn.
